# Type B thymoma in a patient with HIV infection: A case report with a review of HIV and thymoma coexistence

**DOI:** 10.1111/1759-7714.14135

**Published:** 2021-09-05

**Authors:** Yaxuan Zhou, Zhongxing Bing, Yingzhi Qin, Dongjie Ma, Hongsheng Liu

**Affiliations:** ^1^ Department of Thoracic Surgery Peking Union Medical College Hospital, Chinese Academy of Medical Sciences Beijing China; ^2^ Peking Union Medical College MD Program Beijing China

**Keywords:** adaptive immune system, HIV, thymoma

## Abstract

HIV infection predisposes people to cancer, including AIDS‐defining cancers, such as Kaposi sarcoma, and a broad range of non‐AIDS‐defining cancers. Here we report a case with rare coexistence of HIV and thymoma, and summarize all the comorbid cases that currently exist. We found that in all the cases reported, thymoma occurred when CD4+ counts were within a normal range, but the immune response in peripheral T‐cell repertoire remains unknown. In our case, an overview of the immune system under this complicated situation is given for the first time by showing the lymphocyte subpopulations in the blood and the immune cell distribution of the thymoma. This case expands the scope of non‐AIDS‐defining cancers, and provides insight into the influence of the immune system under two immunocompromising conditions, HIV infection and thymoma.

## Graphical abstract

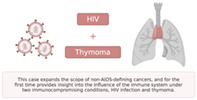

## INTRODUCTION AND BRIEF REVIEW OF HIV AND THYMOMA COEXISTENCE

People with HIV infection are prone to cancer. A broad range of AIDS‐defining cancers (ADCs) and non‐AIDS‐defining cancers (NADCs), including lung cancers, anal cancers, etc., has been documented in HIV‐infected patients.^1^

Thymoma is a rare kind of neoplasm that mostly affects adults aged 40–70 years.^2^ The estimated prevalence of thymoma is 1.3 per million persons in the United States and 3.93 per million in China.^3^, ^4^ Thymoma has been well‐accepted to be correlated with several immune system disorders, such as myasthenia gravis, but its connection with HIV is rarely mentioned. To the best of our knowledge, four prior cases of thymoma with HIV infection have been reported, of which three were well‐described (Table 1).^5^, ^6^, ^7^, ^8^ This comorbidity affected both adults and children, and patients of this kind were speculated to be more prone to opportunistic infections.^7^, ^8^ Together with our patient, several common features are found in these cases. First, thymoma was discovered after HIV in all cases, which fits the definition of HIV‐associated cancer. Second, CD4+ T cells remained at a level much greater than 200/μl the time thymoma was diagnosed. Generally lower CD4+ counts are shown to be associated with higher incidences of ADCs and NADCs, but thymoma occurs when CD4+ counts are preserved.^1^, ^9^

**TABLE 1:** Characteristics of HIV and thymoma coexistence patients

Ref.	Age^a^/sex	Time^b^	Type (WHO)	Stage (Masaoka–Koga)	Treatment	CD4+ T cell (cells/μl)^c^	CD4/CD8 ratio	Complications
6	33/M	22 mo	NR	NR	None^d^	NR	NR	None
7	10/M	10 yr	NR	NR	CT + SR + RT	560	NR	None (after thymoma discovery)
8	44/M	13 yr	Type B2/B3	Stage III	SR + RT + CT	499	1.24	Kaposi sarcoma, osteomyelitis (*Mycobacterium fortuitum*), pulmonary histoplasmosis

Abbreviations: M, male; NR, not reported; CT, chemotherapy; RT, radiation therapy; SR, surgical resection.

^a^ Age refers to the year of thymoma discovery.

^b^ Time refers to period from the diagnosis of HIV to the discovery of thymoma.

^c^ CD4+ T cell was counted after thymoma discovered and before any treatment.

^d^ The patient in reference 6 did not receive surgical resection because the pulmonary structures were invaded and the neoplasm was unable to be resected en bloc.

With these findings, it is crucial to understand how the immune system responds to the coexistence of HIV and thymoma. However, the prior cases focus more on clinical perspectives, with less attention to the abnormality of the immunity index. In our patient, the lymphocyte subpopulations in the blood and immune cell distribution of the thymoma were described, which gave an overview of the immune system under this complicated situation. This case provides insight into the influence of the immune system under the coexistence of HIV infection and thymoma, and the possible role of the thymus in HIV‐positive immunodeficiency.

## CASE REPORT

**FIGURE 1:**
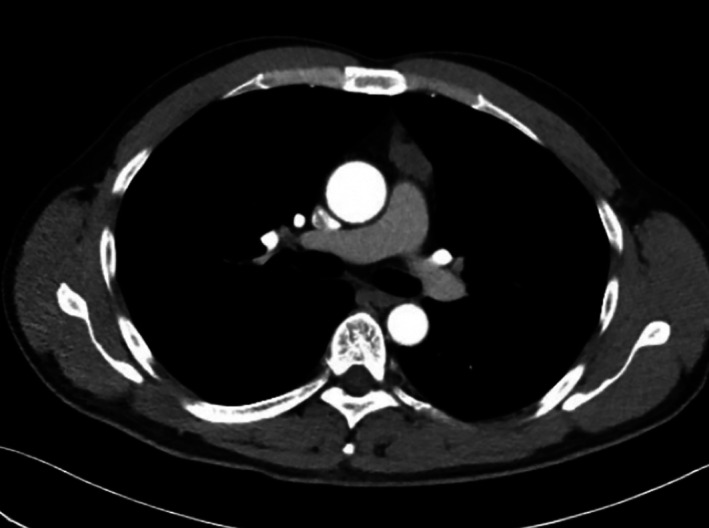
Chest contrast‐enhanced computed tomographic scan in the coronal view shows a soft‐tissue mass located in the anterior mediastinal

A 50‐year‐old Chinese male presented to the hospital in December 2020. A month ago, while he was undergoing a routine physical examination before the surgical excision of his right eye pterygium, a large anterior mediastinal mass was found. A contrast‐enhanced computed tomographic scan revealed a smoothly marginated anterior mediastinal mass measuring 3.1 × 1.4 cm. It was a multi‐lobulated, soft‐tissue mass with slight homogeneous enhancement after intravenous contrast (Figure 1). The patient had no complaint of physical discomfort and denied symptoms of ptosis, diplopia, facial or limb weakness, slurred speech, or dysphagia.

The patient's past history was significant for HIV positivity, radius fracture, and pterygium. HIV infection was diagnosed in April 2018 (probably due to unsafe sex in January 2018) with initial CD4+ T‐cell counts of 480 cells/μl. The patient had been treated with antiretroviral therapy (ART) ever since then, consisting of elvitegravir, cobicistat, emtricitabine, and tenofovir alafenamide fumarate. CD4+ counts elevated to 514 cells/μl in April 2020 and the CD4+/CD8+ ratio was 1.17 at that time. During the course of HIV infection, he reported no history of opportunistic infection or neoplasia. Fracture of his right radius occurred in July 2017 due to high force impact, after which internal fixation was done with implanted plates. Surgery was conducted to excise his right eye pterygium in November 2020 after complaints of visual compromise for 1 year.

**FIGURE 2:**
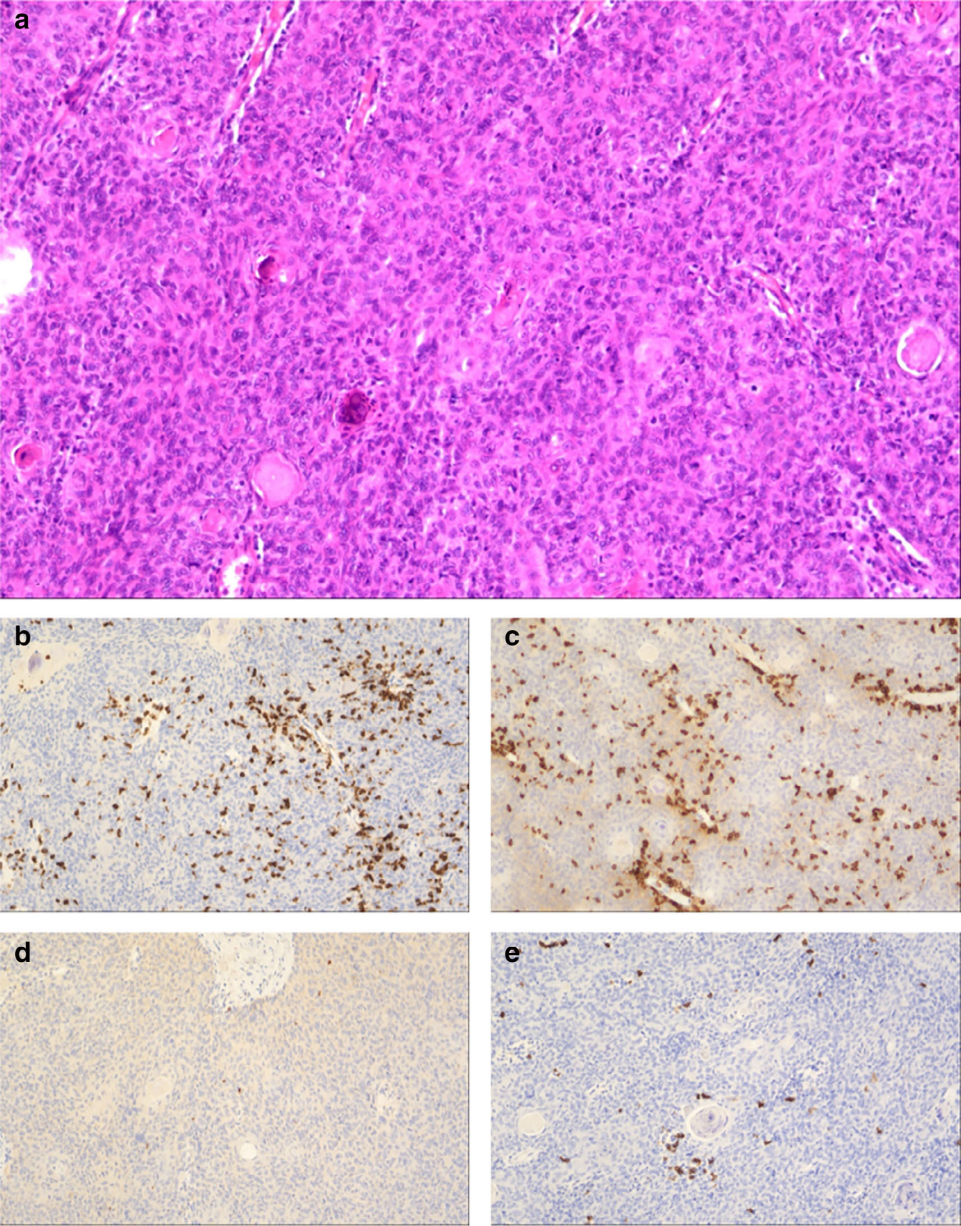
Histology pathology of the patient's thymoma. (a) H&E stain, ×100. (b) Immunohistochemical stain for CD3. (c) Immunohistochemical stain for CD5. (d) Immunohistochemical stain for TdT. (e) Immunohistochemical stain for CD20

Laboratory examination reported negative acetylcholine receptor (AchR) antibodies and normal electromyography. No hepatitis B antigen or antibody was detected. Right before surgery, the patient's leukocyte count and differential lay within the normal range, with a slight increase in monocyte percentage. Complete excision of the lesion with total thymectomy was undertaken by video‐assisted thoracoscopic surgery (VATS). A well‐encapsulated, firm mass of size around 4 × 3 × 2 cm was found in the left thymus. The tumor was stage I in the Masaoka–Koga staging system. Histopathology revealed type B3 (WHO, 2015) thymoma with foci of necrosis. The polygonal cells showed diffuse staining with AE1/AE3 cytokeratin and CD19, and negative for desmin and CD34. Immunohistochemical stains were focally positive for CD3 and CD5, and sporadically positive for terminal deoxynucleotide transferase (TdT) and CD20 (Figure 2).

Right after surgery, the patient's leukocyte counts elevated to 15.74 × 10^9^/L, with the predominance of neutrophils. Lymphocyte dropped to 0.79 × 10^9^/L. Before he was discharged, leukocyte count and differential came back to normal. Chest radiograph showed good lung recruitment with no effusion or gas present. Two weeks after surgery, his lymphocyte subpopulations were as follows: CD4+ T cell = 879 cells/μl, CD4/CD8 = 1.26, CD4 + CD45RA‐/CD4+ = 42.9% (*N* = 57 ± 11.4%), CD4 + CD45RA+/CD4+ = 57.1% (*N* = 43 ± 11.4%), CD4 + CD28+/CD4+ = 100% (*N* = 92.5 ± 7.5%), CD8 + CD28+/CD8+ = 80.5% (*N* = 48.8 ± 11.6%), CD8 + DR+/CD8+ = 17.5% (*N* = 15.05 ± 8.75%), CD8 + CD38+/CD8+ = 27.1% (*N* = 44.9 ± 12.5%). As type B3 thymomas have more probability of recurrence, the patient was suggested for radiation therapy afterwards.

## DISCUSSION

With the widespread use of ART in well‐developed countries, the number of ADC patients has dramatically decreased, while the proportion and absolute number of NADCs in HIV‐associated cancer patients has increased and become the leading cause of death in people with HIV.^1^ By reviewing coexisting cases of HIV and thymoma, we found that thymoma meets the definition and may be a rare type of NADC that has never been mentioned. We have to admit that this coexistence might be accidental, considering that although HIV was diagnosed before thymoma discovery, the occurrence of thymoma may be earlier, and only five cases have been reported.

Another aspect we are concerned about is the immune system under this condition. Thymoma patients are predisposed to immune‐mediated diseases, such as myasthenia gravis and cytopenias. Its pathogenetic model suggests that the abnormal but thymopoietically active intratumorous microenvironment continuously exports mature CD4+ T cells to the blood, gradually replaces normal T‐cell repertoire, and stimulates B cell response even after thymectomy.^10^, ^11^ HIV targets CD4+ T lymphocytes and replicates itself. A previous case reported a HIV and thymoma patient with Kaposi sarcoma and severe opportunistic infections but without T‐cell lymphopenia.^8^ Although our patient has no complications so far, abnormalities in T‐cell subpopulation partially reveal his immune system dysfunction. Same as previously reported cases, a normal CD4+ count and a low CD4+/CD8+ ratio were observed in our patient.^7^, ^8^ A shift from memory/effector to naïve phenotype in CD4+ cells and abnormalities in CD8+ cells were found in our patient's peripheral T‐cell repertoire. We conclude that with two immunocompromising conditions added, absolute CD4+ counts may not reflect the risk faced by patients. Further research is needed to determine how the immune system changes under complicated conditions like this.

## CONFLICT OF INTEREST

The authors declare no conflicts of interest.
